# Wendy Z. Goldman and Donald Filtzer (eds), *Hunger and War: Food Provisioning in the Soviet Union During World War II*

**DOI:** 10.1093/shm/hky036

**Published:** 2018-04-30

**Authors:** Golfo Alexopoulos

**Affiliations:** University of South Florida, Tampa

This excellent edited volume examines the problem of hunger, starvation, nutrition and food supply in the Soviet Union during the Second World War. The book consists of five substantial essays plus an editors’ introduction, an extensive bibliography, index, and list of terms and abbreviations, as well as very helpful and striking charts and tables. The essays include: ‘Introduction: The Politics of Food and War’, by Donald Filtzer and Wendy Goldman; ‘Not By Bread Alone: Food, Workers, and the State’, by Wendy Goldman; ‘The State’s Pot and the Soldier’s Spoon: Rations (Paёk) in the Red Army’, by Brandon Schechter; ‘Queues, Canteens, and the Politics of Location in Diaries of the Leningrad Blockade, 1941–1942’, by Alexis Peri; ‘Nutritional Dystrophy: The Silence and Semantics of Starvation in World War II’, by Rebecca Manley; and ‘Starvation Mortality in Soviet Home-Front Industrial Regions during World War II’, by Donald Filtzer. According to the editors, each chapter sheds light on topics that were once taboo, neglected or forbidden, such as starvation and mortality in the rear. The book is incredibly well documented and researched, and essential reading for anyone who wants to understand the Soviet Union’s wartime experience.

The book makes several important points about the Soviet Union’s wartime management of hunger and its struggle to feed citizens. Wendy Goldman examines the hierarchies of allocation and provisioning. Through a highly differentiated and complicated rationing system, the regime allocated more food to those who expended more calories at work, but it also allocated more food to top managers and skilled workers, as well as workers in priority sectors, such as defence. The state held to certain principles of allocation, taking food from one starving segment of the population in order to feed another, but losing the calorie battle, nonetheless. The editors state that ‘The Red Army benefited considerably from Lend-Lease aid, but only from 1944 on, and Soviet civilians bore the brunt of the shortages created by German occupation’ (p.27).

The discussion is also thoughtful and nuanced. The editors underscore that in order to determine which people were at greater risk of weight loss, nutritional deficiency and starvation, one has to look beyond caloric intake to energy expended. The Soviet wartime rationing system differentiated by age and intensity of labour (for example, Soviet miners doing intense underground work), but it did not account for body mass, which would also dictate an individual’s caloric needs. In general, women had lower rates of mortality than men because they may have required less food than men. Workers, with long workdays and intense labour, who were pushed to produce more during the war were at risk in an environment where food was in short supply. The book highlights the fact that rations alone were insufficient to keep people alive. The government, therefore, encouraged subsidiary agriculture, collective and personal gardens, and collective farm markets. But if people kept themselves alive with potatoes and grains, they suffered from vitamin deficiency and malnutrition. Moreover, the conditions of war and Soviet inefficiency meant that waste, spoilage, theft and problems with food deliveries also diminished overall consumption and meant that people consumed considerably less than the official rations indicated. Thus ‘by 1944, male defense workers, the country’s best-fed civilians, were beginning to die of starvation’ (p.43).

The chapters in this volume are so well done that one is left with the impression that each could and should be expanded into a book. I was especially struck by Rebecca Manley’s examination of nutritional dystrophy. She discusses how during the Second World War, for the first time since the 1920s, hunger and starvation became legitimate objects of medical research. Although the subject of starvation remained highly sensitive and circumscribed, medical researchers found ways to document and conceptualise the widespread effects of hunger on the Soviet population. Manley traces the emergence of a study of nutritional dystrophy, and the fascinating ways in which ‘the blockade generated a new conception of hunger as a unified illness and produced unique conditions for the study of hunger’ (p.219). Hunger became not just a condition of insufficient food, but a medical condition with distinct stages. The ways that Manley traces and deconstructs the Soviet language of hunger and the terms used to describe starvation illnesses is masterful. I read her chapter more than once. It is brilliant.

